# Long-term complete responses after ^131^I-tositumomab therapy for relapsed or refractory indolent non-Hodgkin's lymphoma

**DOI:** 10.1038/sj.bjc.6603166

**Published:** 2006-05-09

**Authors:** F Buchegger, C Antonescu, A Bischof Delaloye, C Helg, T Kovacsovics, M Kosinski, J-P Mach, N Ketterer

**Affiliations:** 1 Service of Nuclear Medicine, University Hospital of Lausanne, CH-1011 Lausanne, Switzerland; 2 Service of Nuclear Medicine, University Hospital of Geneva, CH-1211 Geneva 14, Switzerland; 3 Service of Oncology, University Hospital of Geneva, CH-1211 Geneva 14, Switzerland; 4 Centre for Hematological Malignancies, Oregon Health and Science University, Portland, OR 97239, USA; 5 Institute of Applied Radiophysics, University of Lausanne, CH-1007 Lausanne, Switzerland; 6 Swiss Institute for Experimental Cancer Research, CH-1066 Epalinges, Switzerland; 7 Multidisciplinary Oncology Centre, University Hospital of Lausanne, CH-1011 Lausanne, Switzerland

**Keywords:** radioimmunotherapy, Non-Hodgkin lymphoma, long-term follow-up, ^131^I-tositumomab

## Abstract

We present the long-term results of 18 chemotherapy relapsed indolent (*N*=12) or transformed (*N*=6) NHL patients of a phase II anti-CD20 ^131^I-tositumomab (Bexxar®) therapy study. The biphasic therapy included two injections of 450 mg unlabelled antibody combined with ^131^I-tositumomab once as dosimetric and once as therapeutic activity delivering 75 or 65 cGy whole-body radiation dose to patients with normal or reduced platelet counts, respectively. Two patients were not treated due to disease progression during dosimetry. The overall response rate was 81% in the 16 patients treated, including 50% CR/CRu and 31% PR. Median progression free survival of the 16 patients was 22.5 months. Median overall survival has not been reached after a median observation of 48 months. Median PFS of complete responders (CR/CRu) has not been reached and will be greater than 51 months. Short-term side effects were mainly haematological and transient. Among the relevant long-term side effects, one patient previously treated with CHOP chemotherapy died from secondary myelodysplasia. Four patients developed HAMA. In conclusion, ^131^I-tositumomab RIT demonstrated durable responses especially in those patients who achieved a complete response. Six of eight CR/CRu are ongoing after 46–70 months.

Advanced stage follicular non-Hodgkin's lymphoma (NHL) is considered incurable by conventional therapy, while localized stage 1 or 2 NHL may be cured with external beam radiation therapy of involved sites (Mac Manus and Hoppe, 1996). Current radioimmunotherapy (RIT) is to be considered a combination of systemic radiation therapy and antibody-based immunotherapy. Both treatments reach tumour sites after i.v. application. Each of the two modalities, the unlabelled anti-CD20 antibody therapy and the corresponding RIT have been shown to be effective in follicular NHL (Buchsbaum *et al*, 1992; Kaminski *et al*, 1993; McLaughlin *et al*, 1998; Kaminski *et al*, 2005). The optimal combination of the two therapy strategies might have the potential to cure even advanced stage disease.

Recently, two radiolabelled antibodies targeting the CD20 antigen have been shown to lead to high percentages of complete remissions (Kaminski *et al*, 2000; Witzig *et al*, 2002b). Long lasting durable responses were reported for patients treated with ^131^I-tositumomab either upfront (Press *et al*, 2003; Kaminski *et al*, 2005; Leonard *et al*, 2005) or at recurrence after chemotherapy (Kaminski *et al*, 2000, 2001; Vose *et al*, 2000; Davies *et al*, 2004; Horning *et al*, 2005). A recent pooled analysis of studies in the relapsed/refractory disease setting has demonstrated long-term durable responses lasting 4 years or more associated with a single treatment of ^131^I-tositumomab (Fisher *et al*, 2005). Similar response rates including durable responses were reported for RIT with ^90^Y-ibritumomab tiuxetan (Zevalin®) (Gordon *et al*, 2004b).

Compared with RIT based on ^131^I-labelled antibodies that has to be performed in specially equipped radioprotection units in Europe, using ^90^Y as the label allows outpatient treatment. In the US, ^131^I-tositumomab is administered as an outpatient treatment. The use of ^131^I also has clinical advantages. The low energy electrons are well adapted for treatment of small or minimal disease (Sharkey *et al*, 1991; Vogel *et al*, 1997) and the long experience with ^131^I-therapy of thyroid disease has shown excellent tolerance (Hall *et al*, 1992; Schlumberger and De Vathaire, 1996). Labelling with ^131^I can be performed while completely maintaining antibody immunoreactivity (Schaffland *et al*, 2004). The use of dosimetry for ^131^I-tositumomab allows for patient specific dosing.

Recently, a particularly high percentage of long lasting complete responses was reported by Davies *et al* (2004) in 41 relapsed patients treated in England with ^131^I-tositumomab. As the second European centre participating in this multicentre study, we report here the experience from Switzerland with the particular focus on the long-term efficacy of this ^131^I-RIT.

## PATIENTS AND METHODS

This single arm, open-label phase II study of Corixa Corp., South San Francisco, CA and GlaxoSmithKline, Philadelphia, PA, USA aimed to establish the response rate to ^131^I-tositumomab in patients after first or multiple recurrences of indolent or transformed B-cell lymphoma. Duration of response, safety and survival were secondary end points. The study was conducted in two centres in Switzerland. Patients gave their written informed consent to the study protocol that had been approved by the local Ethics Committees of the University Hospitals of Lausanne and Geneva as well as the Radioprotection Section of the Swiss Federal Office of Public Health.

### Patient eligibility

Patients had to present a histologically confirmed, CD20 positive follicular, small lymphocytic or MALT non-Hodgkin's B-cell lymphoma, in relapse after at least one full regimen of chemotherapy. Patients with transformed NHL were also eligible. Measurable minimal two-dimensional tumour diameter was ⩾2 cm. A HAMA (human anti-mouse antibody) test had to be negative at study entry. Adequate bone marrow function was to be documented with neutrophils ⩾1.5 × 10^9^ l^−1^ and platelets ⩾100 × 10^9^ l^−1^. Maximal acceptable lymphoma infiltration of bone marrow was 25%, as determined with trephine biopsy.

A minimal waiting period of 4 weeks was required after cytotoxic chemotherapy, radiotherapy or cytokine therapy. Patients having had high-dose chemotherapy or radiation therapy including stem cell rescue were excluded from the study as well as patients with known HIV infection, active hydronephrosis, and those treated previously with RIT.

### Dosimetric and therapeutic antibody administrations

Unlabelled and radiolabelled antibody tositumomab was provided by Corixa Corp., South San Francisco, CA and GlaxoSmithKline, Philadelphia, PA, USA. Dosimetric and therapeutic ^131^I-labelling of tositumomab was performed centrally (MDS Nordion Inc., Kanata, Canada) and the labelled compound was shipped frozen on a per patient basis. Two arbitrarily selected therapeutic batches of ^131^I-tositumomab were analysed for immunoreactivity using the Lindmo approach (Lindmo *et al*, 1984). Immunoreactivity was measured immediately after upfreezing the batches for injection of patients. About 6 ng ^131^I-tositumomab were incubated for 2 h at 37°C with five serial dilutions of 1–10 × 10^6^ fresh, exponentially grown Raji or Daudi B lymphoma cells in duplicates. Cell-bound activity was determined and expressed in % of incubated activity, specific binding, after subtraction of nonspecific binding, was determined and expressed as percent of incubated activity and immunoractivity determined using double inverse plot (Lindmo *et al*, 1984) with extrapolation at infinite antigen excess.

Patient preparation included thyroid blocking with 2 × 100 mg KI per day orally and premedication with 500 mg paracetamol and 2 mg clemastine, taken orally. Unlabelled and radiolabelled tositumomab regimen consisted of two steps as described previously (Kaminski *et al*, 1993; Kaminski *et al*, 2005). Shortly, in the first dosimetry step, patients received 450 mg unlabelled tositumomab in a 1 h infusion followed immediately by that of 35 mg ^131^I-tositumomab (185 MBq). The second, therapeutic step consisted again of the infusion of 450 mg unlabelled tositumomab over 1 h followed by that of 35 mg ^131^I-tositumomab labelled with the activity calculated to deliver 75 cGy total body dose for patients with normal, and 65 cGy for patients with reduced platelet counts (100–150 G l^−1^).

For dosimetry, patients were scanned with a large field of view dual head gamma camera (BIAD, Trionix, Twinsburg, Ohio, or Prism 2000, Picker) equipped with high-energy parallel collimators and using a matrix of 1024 × 256. Camera uniformity, background and energy peaks were checked daily. Scanning was performed as described by Wahl *et al* (1998). Patients were scanned under identical conditions, on day 0 immediately after antibody perfusion (referred to as 100% reference scan), on day 2, 3 or 4 and on day 6 or 7. The background corrected activities of days 2, 3 or 4 and day 6 or 7 were expressed in % of activity measured on day 0 and fitted to a semi-logarithmic scale allowing to directly read, from the exponential curve-fit, the whole-body effective half-life and retention time of ^131^I-labelled tositumomab. Using the published tabulated whole-body dosimetry data (Wahl *et al*, 1998), the ^131^I-tositumomab activity was extrapolated that delivered 75 cGy to patients with normal BM function or 65 cGy to patients with reduced platelet counts.

### Response evaluation

Tumour response evaluation, including physical examination, computed tomography (CT) of the neck, chest, abdomen and pelvis and bone marrow biopsy if involved before treatment, was performed at weeks 13 and 26 and every 6 months thereafter until disease progression or death. A complete response (CR) was defined as complete resolution of all disease-related radiologic abnormalities and clinical symptoms. An unconfirmed CR (CRu) was defined as complete resolution of clinical disease symptoms and residual focal stable radiologic abnormalities of ⩽2 cm diameter according to the International Workshop criteria (Cheson *et al*, 1999). A partial response (PR) was defined as >50% reduction of two-dimensional tumour size and progressive disease as ⩾25% increase of the two-dimensional tumour size. Progression free survival (PFS) was defined as time from treatment initiation to first documentation of progression or death and overall survival (OS) as time from treatment start to death.

### Evaluation of toxicity and safety

All adverse events from study entry through to week 13 were graded according to the National Cancer Institute Common toxicity criteria. Adverse events after this period considered to be possibly or probably related with study drug were also recorded. Detailed blood analysis was performed weekly from study week 3–9 or until recovery and repeated at 13 and 26 weeks and every 6 months thereafter until 2 years. Other toxicity evaluations included blood chemistry and TSH assays for all patients basically all 6 months up to 2 years. Long-term evaluations after 2 years from treatment were performed every 6 months.

### HAMA assay

The HAMA assay (HAMA Elisa, Medac, Hamburg, Germany) was performed for all patients and had to be negative at inclusion. The HAMA assay was repeated at 7, 13 and 26 weeks after therapy and every 6 months thereafter.

### Statistical analysis

Statistical analysis was performed for patients who had received the therapeutic activity of radiolabelled antibody. PFS and OS were analysed using the Kaplan Meier analysis of the UNISTAT 5.5® statistical package for Windows (2002 edition, Unistat Ltd, London, England, run on Windows XP). Survival differences among patients subgroups were analysed using the Wilcoxon *χ*^2^ statistics (Gehan evaluation). *P*-values <0.05 were considered significant.

## RESULTS

In all, 18 patients were included in the study. Their characteristics and their lymphoma history are shown in Table 1. Two patients with transformed disease did not receive the administration of therapeutic ^131^I-tositumomab activity because of rapid disease progression during the dosimetry phase.

As concerning chemotherapy regimens, the eight patients experiencing CR after RIT had a mean of 2.5 chemo/rituximab treatments and one patient had RT before inclusion. In detail, 88% of these patients had received anthracyclines, 75% alkylating agents and 63% rituximab. The eight patients experiencing PR or PD after RIT had a mean of 3.4 chemo/rituximab treatments and two patients had RT before inclusion. In detail, all of these patients had been treated with both anthracyclines and alkylating agents and 75% with rituximab.

### Pharmacokinetics

**Figure 1:**
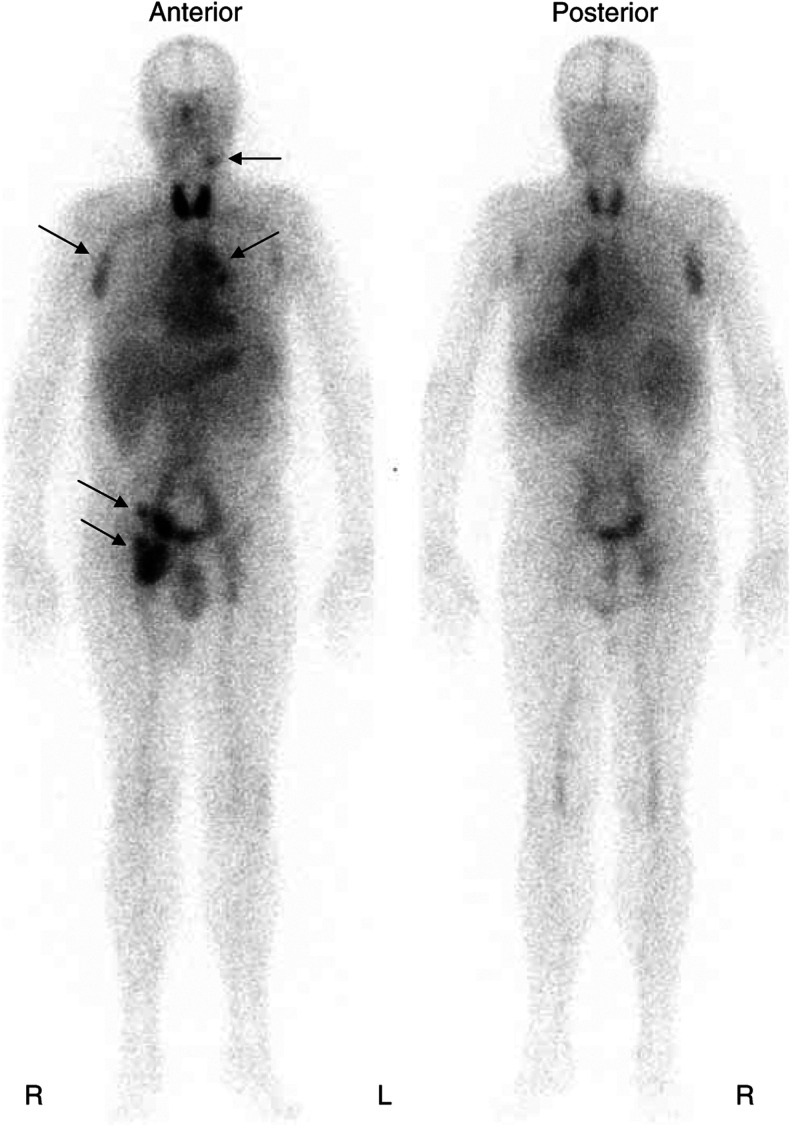
Anterior and posterior whole-body scans of patient Corixa Nr. 721 are shown recorded 6 days after injection of therapeutic ^131^I-tositumomab activity (3.0 GBq). Antibody uptake is clearly visualized (arrows) in the left jugular, right axillar, left mediastinal, right iliac and right inguinal adenopathies. R=right, L=left. Thyroid uptake of ^131^I was significant in this patient despite thyroid blockade with oral KI. Six days after injection, blood pool activity remains high.

After the injection of the dosimetric activity of 185 MBq ^131^I-tositumomab, the whole-body scintigraphies revealed a single exponential radioactivity decrease in all patients with a mean effective half-life of 64.2±8.7 h was observed (range 47.9–77.8 h). An average therapeutic activity of 2.9±0.6 GBq (range 1.9–3.9 GBq) ^131^I-tositumomab was administered after a mean delay of 8 days after dosimetry (Figure 1). Immunoreactivity of two arbitrarily selected therapeutic batches of ^131^I-tositumomab revealed as very high at extrapolation to infinite antigen excess with results ranging between 95.2 and 100%.

### Response and survival

**Figure 2:**
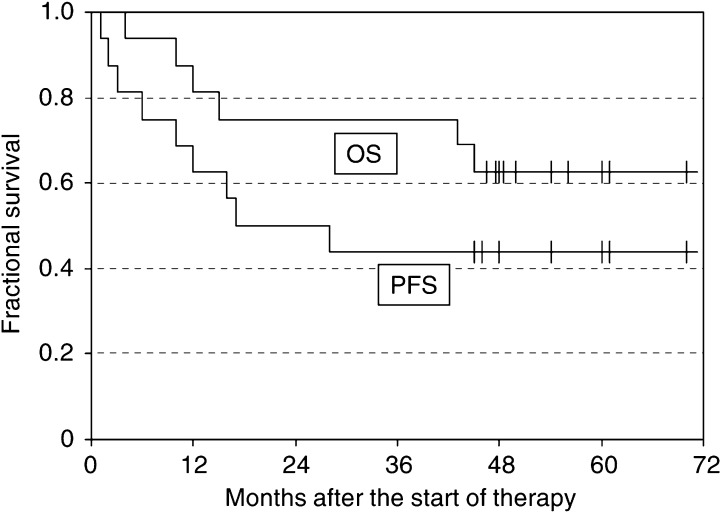
Kaplan–Meier plots show OS and PFS of the 16 treated patients, 12 presenting with indolent relapsed lymphoma and four with transformed disease.

The overall response rate was 81% in the 16 patients treated, including 50% CR/CRu and 31% PR. Median PFS of the 16 treated patients was 22.5 months. Median overall survival has not been reached after a median observation time of 48 months (Figure 2).

**Figure 3:**
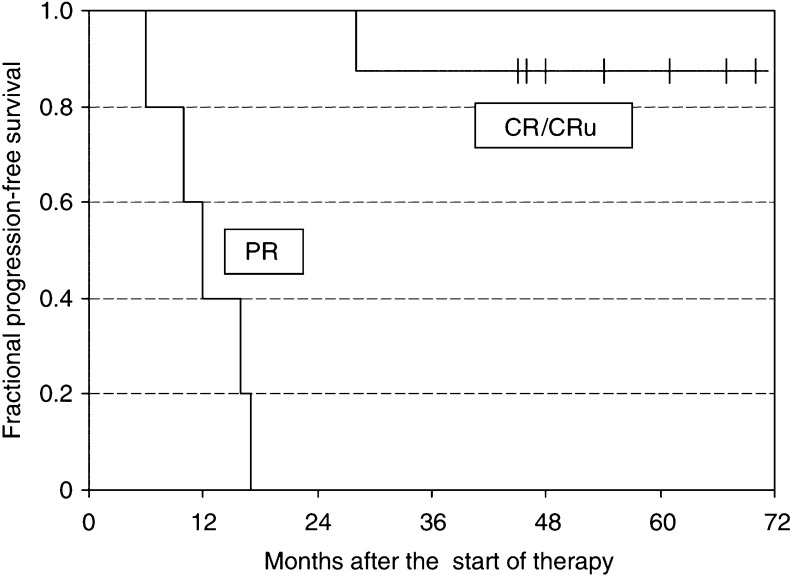
Kaplan–Meier plots show PFS of 13 responding patients, 12 presenting with relapsed indolent lymphoma and one with transformed disease. Results are shown according to the response, PR or CR/CRu. Note that of the eight patients with CR/CRu, one patient has relapsed at 28 months and one patient died at 45 months from secondary leukaemia, the other six patients are in ongoing CR/CRu.

All 12 patients with indolent lymphoma responded, eight with CR or CRu and four with PR. After a median follow-up of 45.5 months, the median PFS of the 12 indolent lymphoma patients has not been reached. The PFS of the four patients in PR ranged from 6 to 17 months. Median PFS of complete responders (CR and CRu) has not been reached but will be greater than 51 months (Figure 3). In fact, only one of the eight CR/CRu patients presented a relapse at 28 months. Another patient in CRu died from secondary myelodysplastic syndrome (MDS) and leukaemia 45 months after RIT, as described below. Six patients remain currently in ongoing CR/CRu, two of them being disease free 4 years after therapy and three others after more than 5 years. These six patients had previously been treated with a mean of 2.7 chemo-immunotherapy regimens (range 1–5), two of them had also received radiotherapy.

Of the five patients treated with transformed disease, one had a partial response that lasted 10 months. The three other patients progressed and required further treatment before treatment-related haematological toxicity was resolved.

*χ*^2^ Wilcoxon analysis indicated a highly significant difference (*P*<0.005) of PFS in patients with non-transformed (*N*=12) compared with transformed disease (*N*=4). However, given the very low number of patients with transformed disease who actually received the RIT, this statistical comparison must be considered with caution. Other parameters were evaluated for patients with non-transformed disease (*N*=12). The analysis indicated that stage of disease, bone marrow involvement, tumour size, age, gender, number of previous chemotherapies, platelet counts, LDH level, and time from initial diagnosis were not significantly linked with treatment outcome (*P*>0.25).

### Toxicity

During first perfusion of unlabelled tositumomab, one patient had a fall of blood pressure that required adjustment of the infusion rate and another patient developed grade 2 abdominal pain requiring opioid treatment.

All 16 treated patients showed either enhanced pre-existing or newly developing bone marrow depression in at least two cell lineages (Table 2). Bone marrow depression was minor (grade 1 or 2) in four patients. The other 12 patients showed grade 3 (five patients) or 4 (seven patients) toxicity in one or more lineages. The median degree of PMN, leukocyte and platelet depression after RIT was grade 3, that of haemoglobin grade 1. The median duration of toxicity greater than grade 2 was 21–27 (Table 2). One patient had the leukocyte and PMN nadir particularly late and was recovering from these grade 3 and 4 toxicities, respectively, after week 13. Another patient with transformed disease required further treatment 7 weeks after RIT because of disease progression while still in grade 3 haematological toxicity. Some minor toxicities grade 1 or 2 were still observed in the other 14 patients 13 weeks post-treatment.

In all, 29 nonhaematologic side effects, mostly mild (grade 1 or 2) possibly or probably linked with RIT, were reported for nine patients. They included pain, fatigue, fever, chills, sweating, rash, arthralgia, erythaema and oedema, a pneumonia and a thyroiditis. One febrile lymphadenitis during pancytopenia and one febrile neutropenia, both grade 3, were observed at 7 and 8 weeks post RIT and resolved both rapidly under antibiotic therapy. One patient developed elevated TSH without requiring hormone replacement.

Four patients developed HAMA. Interestingly, all positive HAMA results were observed among the patients who responded with CR/CRu, suggestive of a significant association (*P*< 0.05) in a multigroup comparison according to Tukey-HSD. The four HAMA positive patients are still alive without relapse.

The most serious adverse event was a myelodysplastic syndrome (MDS) that appeared 34 months after RIT in one patient who eventually died from secondary leukæmia. However, this patient was previously treated with five cycles of chemotherapy (CHOP), with which he reached a PR. At progression of disease, 19 months later, he was treated with rituximab, but a major para-aortic mass showed continued growth. He was included in the RIT study 40 months after initial diagnosis and initial CHOP chemotherapy and he presented a bulky abdominal mass. RIT with 3.6 GBq ^131^I-tositumomab resulted in CRu without further change over the rest of follow-up (stable CT over more than 2 years). MDS was diagnosed 34 months after RIT and 74 months after initial chemotherapy. It evolved into secondary leukaemia with fatal outcome 11 months later.

## DISCUSSION

Results of 16 patients treated with ^131^I-tositumomab RIT for relapsed indolent or transformed lymphoma are presented. The long-term results in low-grade disease are very encouraging, considering that 6 out of 12 patients are disease free after 46 to 70 months.

The initial phase I-II studies with ^131^I-tositumomab reported CR in approximately one third of the patients, with half of them relapsing within 20 months (Kaminski *et al*, 2000). The CR rate was 20% in the pivotal study, and half of these complete responders remained disease free for more than 4 years (Kaminski *et al*, 2001). A more recent report (Davies *et al*, 2004) showed 20 CR/CRu (49%) out of 41 patients treated, with a median duration of the CR/CRu not yet reached, but will exceed 2.5 years. Our results compare well with these previous studies, showing 50% CR/CRu, the median PFS for these patients being not reached after a median follow-up of >4 years. Such an outcome is unusual in this population of largely pretreated patients, considering that the natural history of indolent lymphomas is typically characterized by continuous relapses with remissions becoming shorter after each progression (Gallagher *et al*, 1986). It is impressive to note that two of the patients who were in relapse after 4 or 5 lines of chemo-immunotherapy remain in CR 61 and 67 months following RIT.

The impressive efficacy of ^131^I-tositumomab in some patients with chemotherapy resistant disease, would suggest that RIT should be used earlier in the treatment course of indolent lymphoma for optimal clinical benefit. To explore this hypothesis, a recent analysis of more than 1000 patients was conducted to examine the efficacy of ^131^I-tositumomab by line of therapy. This analysis revealed that both the frequency of long-term durable responders and the rate of CR was higher when ^131^I-tositumomab was used earlier in the treatment sequence (Gregory *et al*, 2005). Interestin*g* results have also been reported for follicular lymphoma treated upfront with a single treatment of ^131^I-tositumomab (Kaminski *et al*, 2005). In this latter study, CR were observed in 75% of patients, relapse free survival of these patients was 77% at 5 years. Impressive results have also been observed when ^131^I-tositumomab has been combined with chemotherapy in the upfront setting (Press *et al*, 2003; Leonard *et al*, 2005). Rituximab has been shown to improve the outcome of patients with previously untreated follicular lymphoma when associated with chemotherapy (Marcus *et al*, 2005). Several other reports suggest that first line combination of rituximab and anthracycline-containing chemotherapy is able to induce very high response rates and prolonged remission, including molecular remission (Hiddemann *et al*, 2003; Salles *et al*, 2004; Zinzani *et al*, 2004; Czuczman *et al*, 2005). Both chemo-immunotherapy and RIT appear as attractive options for treatment of indolent lymphoma, but the best long-term strategy still needs to be determined. A randomized phase III study by the SWOG comparing R-CHOP to ^131^I-tositumomab-CHOP is currently ongoing and will address the question of the optimal treatment in the front-line setting.

The results published by Davies *et al* (2004) and our data suggest that ^131^I-tositumomab RIT is at least equivalent to ^90^Y-ibritumomab tiuxetan (Zevalin®) (Gordon *et al*, 2004b). Indeed, in the rituximab refractory setting, efficacy may be more favourable for ^131^I-tositumomab when reviewing two separate phase II studies of both these agents. The PFS for responders treated with ^131^I-tositumomab was 24.5 months *vs* 8.7 months for similar patients treated with ^90^Y-ibritumomab tiuxetan (Witzig *et al*, 2002a; Horning *et al*, 2005). This observation may be explained by the fact that the unlabelled antibody used with ibritumomab tiuxetan is rituximab, which was being administered to patients who were already resistant to rituximab. The efficacy results of ^131^I-tositumomab also compared favourably with ^90^Y-ibritumomab tiuxetan in two separate recent analyses of the long-term durable responder patient populations (defined as time to progression of ⩾12 months) treated with both these agents (Fisher *et al*, 2005; Wiseman and Witzig, 2005). Compared with rituximab in a randomized phase III study, ^90^Y-ibritumomab tiuxetan was shown to induce 34% of CR/CRu *vs* 20% for rituximab and a median time to progression (TTP) of 15 versus 10.2 months. In patients who achieved CR/CRu TTP was 24.7 months for RIT and 13.2 months for rituximab alone (Gordon *et al*, 2004b). Other phase II trials with ^90^Y-ibritumomab tiuxetan reported CR/Cru rates between 15 and 51% (Wiseman *et al*, 2002; Witzig *et al*, 2002a; Gordon *et al*, 2004a) the follow-up being frequently shorter compared with the present study or that of Davies (Davies *et al*, 2004). Therefore, it is clear that both agents have demonstrated high levels of efficacy in patients with relapsed/refractory disease.

In the present study, we observed a single patient who developed secondary myelodysplasia 34 months after RIT and 74 months after first-line chemotherapy. The standard CHOP chemotherapy or/and ^131^I-tositumomab RIT may have contributed to this evolution. Both the alkylating agents and doxorubicin, constituents of CHOP, are known to induce increased risk of myelodysplasia and secondary leukaemia, as does also total body irradiation (Armitage *et al*, 2003). The risk of MDS seems to be particularly increased when radiotherapy (RT) is associated with alkylating agents. It is globally estimated that 10% of patients treated for lymphoma will develop secondary MDS/AML within 10 years of primary therapy (Armitage *et al*, 2003). Thus, the myelodysplasia developing 74 months after initial therapy in a single patient does not allow to conclude on the role of RIT in the course of the illness of this patient. It is also noted that no secondary myelodysplasia was observed in the other report from the English group using the same treatment protocol (Davies *et al*, 2004), and that large cohort studies analysing patients treated with RIT did not show any obvious increase in the incidence of secondary MDS/AML, compared with similar patients treated with conventional therapies (Witzig *et al*, 2003; Bennett *et al*, 2005). In addition, when ^131^I-tositumomab was administered as a single agent in the front-line setting, no cases of MDS/AML were observed after 5 years of follow-up (Kaminski *et al*, 2005).

Both ^131^I-tositumomab and ^90^Y-ibritumomab tiuxetan RIT consist of a combination of radiolabelled with unlabelled antibody. The unlabelled antibody, however, might not be used optimally. A randomized study performed in patients with follicular lymphoma has shown that different schedules using rituximab in maintenance following the standard 4 weeks treatment were significantly improving the event free survival of responders (Ghielmini *et al*, 2004). As RIT is given in a similar patient population, higher overall and complete response rates and longer event-free survival might possibly be achieved by using prolonged treatment with unlabelled antibody following RIT administration.

Another remark concerns the half-life of ^131^I-tositumomab. It has been argued that a long-lived radiolabelled antibody might induce bone marrow and normal tissue toxicity, whereby limiting the amount of activity that can be administered, but there have never been any published clinical data reported to support this theory, to our knowledge. In the 16 patients treated here, ^131^I-tositumomab showed a mean whole-body half-live of 64.2 h. In comparison, ^90^Y-Zevalin was reported to have a median blood effective half-live of 27 h (Wiseman *et al*, 2003). The observed efficacy of ^131^I-tositumomab rather argues against the hypothesis that antibodies labelled with longer-lived radionuclides are potentially more harmful than those with shorter-lived radionuclides. Instead, one might argue that the observed efficacy of ^131^I-tositumomab could be linked with the prolongation of therapeutic radiation delivery over several days, allowing therefore repair, repopulation, redistribution and reoxygenation processes to occur in tumour and normal tissues, as it is the case in standard fractionation radiotherapy (Thames and Hendry, 1987).

A final comment concerns the observation of HAMA in the group of CR patients with long-term survival. Indeed, appearance of HAMA could potentially also induce anti-HAMA antibodies, which could in turn be anti-idiotype antibodies and therefore directed against CD20. Since anti-CD20 antibodies are efficient as lymphoma therapy, anti-idiotype antibodies developed by the patient could represent a long-term treatment to lymphoma provided by the patient himself. However, since tositumomab is a mouse antibody, the HAMA response most probably represents an anti-constant domain antibody and not an anti-idiotype. The correlation of positive HAMA in connection with long-term tumour-free survival might, therefore, rather reflect a preserved immune competence of these patients. Preserved immune competence could be decisive in connection with the use of the unlabelled antibodies that develop efficacy based probably on ADCC and CDC as shown by different studies. Interestingly, a recent report, published while this article was reviewed (Azinovic *et al*, 2006), also described a survival benefit associated with HAMA.

### CONCLUSION

Our data have shown high overall and complete response rates combined with the observation of long-term progression free survivals of CR/CRu in recurrent indolent NHL. Toxicity was mild except in one patient who developed a secondary myelodysplastic syndrome that evolved into secondary leukaemia. However, this observation does not allow any particular deduction at this time on the part of RIT in the cause of this disease. Overall, these data confirm and extend the previously reported results with ^131^I-tositumomab, a treatment shown to be able to bring a significant number of indolent, relapsed NHL into long-lasting complete responses. Our findings in patients at first or second relapse, along with other published reports, also suggest that ^131^I-tositumomab should be administered early in the course of relapsed disease for optimal clinical benefit.

## Figures and Tables

**Table 1:** Patient characteristics (*N*=18)

*Age (years)*	
Median	52.3
Range	31–77
	
*Sex*	
Male	*N*=8
Female	*N*=10
	
*Lymphoma histology*	
Follicular	*N*=10
Small lymphocytic	*N*=1
Malt	*N*=1
Transformed	*N*=6
	
*Stage at study entry*	
II	*N*=3
III	*N*=4
IV	*N*=11
Bulky tumour (largest diameter⩾5 cm)	*N*=14
Bone marrow involvement	*N*=7
	
*Disease evolution from diagnosis to study entry (months)*	
Median	31.2
Mean	45.1
Range	8–121
	
*Prior chemo-rituximab therapies*	
Mean	3.0
Range	1–6
	
Previous RT	*N*=4
Previous rituximab	*N*=10
Elevated LDH	*N*=7
Reduced platelet counts	*N*=4

**Table 2:** Haematological toxicity in 16 treated patients

	**WBC**	**Neutrophils**	**Platelets**	**Hæmoglobin**
Median nadir value (Counts mm^−3^ or g/l, respectively)	1900	600	29 000	109
Median time to nadir (days)	47.5	49	41	58
Range	35–81	35–81	35–62	41–76
Median duration of toxicity grade 3 or higher (days)	20.5	27	26	
Range	8–56	7–49	6–61	(21 & 26)^a^
				
*Toxicity (*%*)*				
Grade 3 or 4	69	69	56	13
Grade 4	19	38	6	Ø

a Only two patients had haemoglobin toxicity grade 3. Duration was 21 and 26 days, respectively.
